# Voice of the Customer Videos: An Educational Tool to Identify Unmet Clinical Needs and Develop Empathy for Medical Device Users

**DOI:** 10.1007/s43683-025-00206-5

**Published:** 2025-12-02

**Authors:** Sarah Ilkhanipour Rooney, Shameeka M. Jelenewicz

**Affiliations:** 1 Department of Biomedical Engineering, University of Delaware, Newark, DE USA; 2 Center for Research in Education & Social Policy, University of Delaware, Newark, DE USA

**Keywords:** Clinical needs, Design, Empathy, Medical devices, Student engagement, Biomedical engineering education

## Abstract

**Purpose:**

Empathy and incorporation of the voice of the customer (VoC) are important elements of the medical device design process, particularly when defining unmet needs and design criteria. However, limited approaches, beyond clinical immersion, have been described to teach biomedical engineering students these skills. Clinical immersion programs struggle with scalability. Our goals are to help students learn, through an accessible format, how to identify unmet clinical needs and develop design inputs that consider user needs, increase students’ empathy for users of medical devices, and foster course engagement.

**Methods:**

To introduce biomedical engineering students to VoC in a scalable way, we recorded interviews with patients, clinicians, and researchers who use medical devices. We refer to these interviews as “VoC videos.” In this paper, we describe our process to create these VoC videos. We measured their efficacy through direct assessments of student work, pre- and post-course survey data, and focus groups with students.

**Results:**

37 VoC videos have been created and used across multiple years of a biomedical engineering course. We found that 1) students can use the VoC videos to inform their development of need statements and design inputs, 2) the VoC videos help students develop empathy for users of medical devices, and 3) the VoC videos foster engagement in course content.

**Conclusion:**

The VoC videos serve as an effective educational tool to support student engagement, empathy, and design skills. The videos are available online for others to use, demonstrating scalability.

**Supplementary Information:**

The online version contains supplementary material available at 10.1007/s43683-025-00206-5.

## Introduction

Practicing biomedical engineers must identify unmet needs and define design inputs for medical devices. The National Center for O*NET Development rates “Operations Analysis—Analyzing needs and product requirements to create a design” at an importance level of 69/100 for biomedical engineers, which is the third highest skill importance score [1]. Understanding the perspectives of medical device users is a crucial element in this process and requires deliberate training and practice.

### Voice of the Customer (VoC)

Voice of the Customer (VoC) is the practice of collecting user feedback to inform products and services. It is an important component of user-centered design regularly implemented in the medical devices industry [2, 3]. VoC can be attained through interviews, focus groups, observations, and ethnographic research. Users typically include patients and clinicians and sometimes payers [3]. Although there are a number of stakeholders in medical device design in addition to patients and clinicians, such as the business (executives, marketing, sales representatives, reimbursement), R&D teams, and external vendors, input from these stakeholders may instead be captured through voice of the business or voice of the technology [3].

For each strategy used to collect VoC, one may consider “capture” and “synthesis/analysis” stages. The “capture” stage focuses on how to collect the highest quality, unbiased information from the appropriate sources. The “synthesis/analysis” stage evaluates the raw data that was collected for common patterns and compares these themes between sources. This stage may lead back to more capture in a cyclic process. VoC is important for all phases of the front-end product development process, including discovery, prototyping, and concept validation [3]. In this paper, we focus on VoC captured through interviews during the discovery phase, leading to development of a need statement and functional requirements, similar to [2].

Given that user perspectives are vital in informing medical device design, it is important that we deliberately train biomedical engineering undergraduates in this skill. In fact, prior work has recommended that manufacturers be trained on the use and benefits of human factors engineering methods in the medical device design and development process [4]. Bringing this education even earlier, into the undergraduate academic setting, would help prepare graduates with valuable skills to apply in the medical devices industry. Specifically, we intend to train students with skills in needs-finding and requirements definitions.

### Clinical Immersion Programs

The most widely discussed way to instruct students in identifying clinical needs is through clinical immersion programs. The primary objective of clinical immersion programs is to identify clinical needs, typically accomplished through student observations of doctors, nurses, and other clinical staff [5]. Given the emphasis on conducting primary observations and interviews, the “capture” stage of VoC is an important focus of clinical immersion programs, and many programs include lessons centered around such topics. In addition to identifying unmet clinical needs, some clinical immersion programs have reported student outcomes of improved critical thinking and problem-solving skills, empathy, motivation, and knowledge of procedural medicine [5]. Furthermore, although needs finding is typically described as the focus of clinical immersion programs, resulting in a written need statement, stakeholder input is also critical in defining design criteria. Publications describing clinical immersion programs do not typically explain how students may be implementing the feedback of medical device users to create design inputs (functional and design requirements), in addition to writing a need statement.

Despite their touted benefits, clinical immersion programs are inherently difficult to scale, with participation averaging less than 50% of the eligible student population [5], posing an issue of accessibility and equity. These programs are limited by the number of available sites/clinicians, costs due to when the course or program is offered (e.g., during off-cycle semesters, such as winter or summer special sessions), scheduling and transportation needs, and the sometimes extensive clearance process for students to enter a clinical setting [6]. The dominating challenge from the administrative side is finding clinical partners [5]. Furthermore, some programs restrict participation to only upper-level students to ensure adequate preparation, maturity [5–7], and motivation for senior design. If needs-finding skills are not introduced or developed elsewhere in the curriculum, then the low and often late (junior or senior year) participation in clinical immersion programs means that the majority of students are unable to engage in repeated practice, a critical component of skill development [8], to strengthen their ability to identify unmet clinical needs. Therefore, there needs to be a more accessible way for students to develop needs-finding skills in addition to clinical immersion programs.

### Virtual Immersions and Videos

To address this challenge of accessibility, others have developed simulated and virtual clinical immersion programs. Medical simulation labs are valuable educational tools and have been used successfully to train students in needs finding [9]; however, these facilities are not available at all institutions. Combined with an in-person clinical immersion program at Clemson University, students video interviewed clinicians on proper techniques for procedures and problems that the clinicians encountered [10]. Students found these clinician video interviews to be the most useful of the activities in the program [10]. Mittal et al. describe how student teams in a “Clinical Needs Finding Video Internship” created 15-minute, documentary-style videos from their in-person clinical rotations [11]. The nine videos recorded key observations in the medical environment and were edited to remove bias toward solutions and instead allow the viewers to focus on needs-finding [11]. One video was used as an instructional aid in a subsequent course, where senior-level student viewers successfully used the video to identify unmet clinical needs [11]. Similarly, students in a different program watched three stakeholder interviews and used them to create personas and make changes to design requirements [12]. In another example, Brennan-Pierce et al. pivoted to a virtual clinical immersion program in response to the COVID-19 pandemic [13, 14]. Students participated in online meetings with clinicians and medical device sales representatives, which included videos of procedures, tours of hospital areas, presentations, and opportunities for questions and answers [13]. As a result of participating in the program, students reported gains in incorporating user needs into design solutions [14]. The virtual format increased accessibility, but there was less breadth and fewer senior capstone project ideas generated [13]. Despite this, some viable projects were carried forward, indicating that students were able to successfully identify some unmet clinical needs through the experience [13]. A Virtual Clinical Immersion Library was created based on the program and used by senior design students in an assignment documenting perceived clinical needs [14]. To bridge a 2D and 3D experience, a virtual reality clinical immersion program was developed by King and Salvo, supplemented by additional video interviews [15]. They found the 3D perspective to be more immersive and increase a sense of presence for the students compared to traditional 2D videos [15].

Videos have also been used outside of biomedical engineering to collect the experiences of individuals interacting with the healthcare system. For example, a prior study determined that YouTube content created by caregivers of stroke survivors offered insight into the caregivers’ unmet needs [16]. Patient Voices (https://www.patientvoices.org.uk/), a catalog of reflective digital storytelling, is another example that captures narratives from patients, caregivers, and professionals. These short (2–4 minutes), compelling videos focus on first-person stories, but they generally do not address the equipment or medical devices used, lacking the direct bridge to engineering design, and it can be challenging to find multiple clinical and patient perspectives on the same topic.

Overall, although these various videos and programs have demonstrated promising initial success in providing scalable opportunities for students to develop skills in needs finding, they focus more generally on the clinical environment than on specific medical devices, reducing their usefulness for generating design inputs for a new solution. Additionally, there has been no evaluation on whether the videos help students develop empathy for users of medical devices.

### Empathy

In research, empathy has been defined in numerous ways. Cuff et al. attempted to synthesize these definitions by recognizing that empathy entails perceiving and understanding the experiences and resulting emotions of others [17]. The concept of empathy is crucial for the successful design of medical devices. Andres and Juanita explain that it is “a fundamental skill for designers to acquire an in-depth understanding of people (i.e., end-users and other stakeholders) so that products, services, environments, systems, and experiences meet human needs, expectations, and aspirations” [18]. Empathic design centers the user throughout the process [18]. Additionally, biomedical engineering faculty view empathy as a vital skill that biomedical engineering students should develop for not only the success of design solutions but also personal and professional development [19]. Empathy is expected to improve communication, intercultural awareness, value created, and motivation for action [19]. Faculty have reported that empathy contributes to discovery of unmet needs, problem solving to address these needs, and creating value [19].

Biomedical engineering faculty in one study believe that empathy is a teachable skill [19], and other studies have demonstrated this to be true. This skill can be cultivated through narratives and creative arts [20], case studies [20], role-playing [21], reflections on animal subjects research [22], in-class modules [23, 24], stakeholder engagement and immersion [20], and a multi-semester training program with interactive exercises and interactions with people with disabilities [25]. Interviews with stakeholders are also believed to help develop empathy [12, 19].

Lastly, interest in “helping others” positively predicts interest in the biomedical engineering major [26]. Furthermore, biomedical engineering is perceived as the most empathetic engineering discipline [27]. These findings suggest that biomedical engineering students may be drawn to opportunities that support empathy. Based on this, we suspect that a user-centered approach would support student engagement in course content.

### Goals and Research Questions

To summarize, empathy and incorporation of the voice of the customer (VoC) are important elements of the medical device design process, particularly when defining unmet needs and design criteria. However, limited approaches, beyond clinical immersion, have been described to teach biomedical engineering students these skills. Clinical immersion programs struggle with scalability. Video recordings have demonstrated preliminary success but with limited evaluation.

To address these gaps, our goals are to help students learn, through an accessible format, how to identify unmet clinical needs and develop design inputs that consider user needs, increase students’ empathy for users of medical devices, and foster course engagement. To introduce biomedical engineering students to VoC in a scalable way, we recorded interviews with patients, clinicians, and researchers who use medical devices. We refer to these interviews as “VoC videos.”

In this paper, we describe our process to create these VoC videos and then address our research questions:

- **RQ1:** Are students able to use VoC videos to inform their development of need statements and design inputs?
- **RQ2:** Do recorded VoC videos help students develop empathy for users of medical devices?
- **RQ3:** Do recorded VOC videos foster engagement in course content?

## Methods

We created VoC videos to bring the voice of the customer to the student, broadening student participation compared to a traditional clinical immersion program. In this section, we describe the process used to develop the VoC videos, an overview of the context in which they were implemented in a course, and the methods used to assess the research questions.

### Developing VoC Videos

**Fig. 1:**
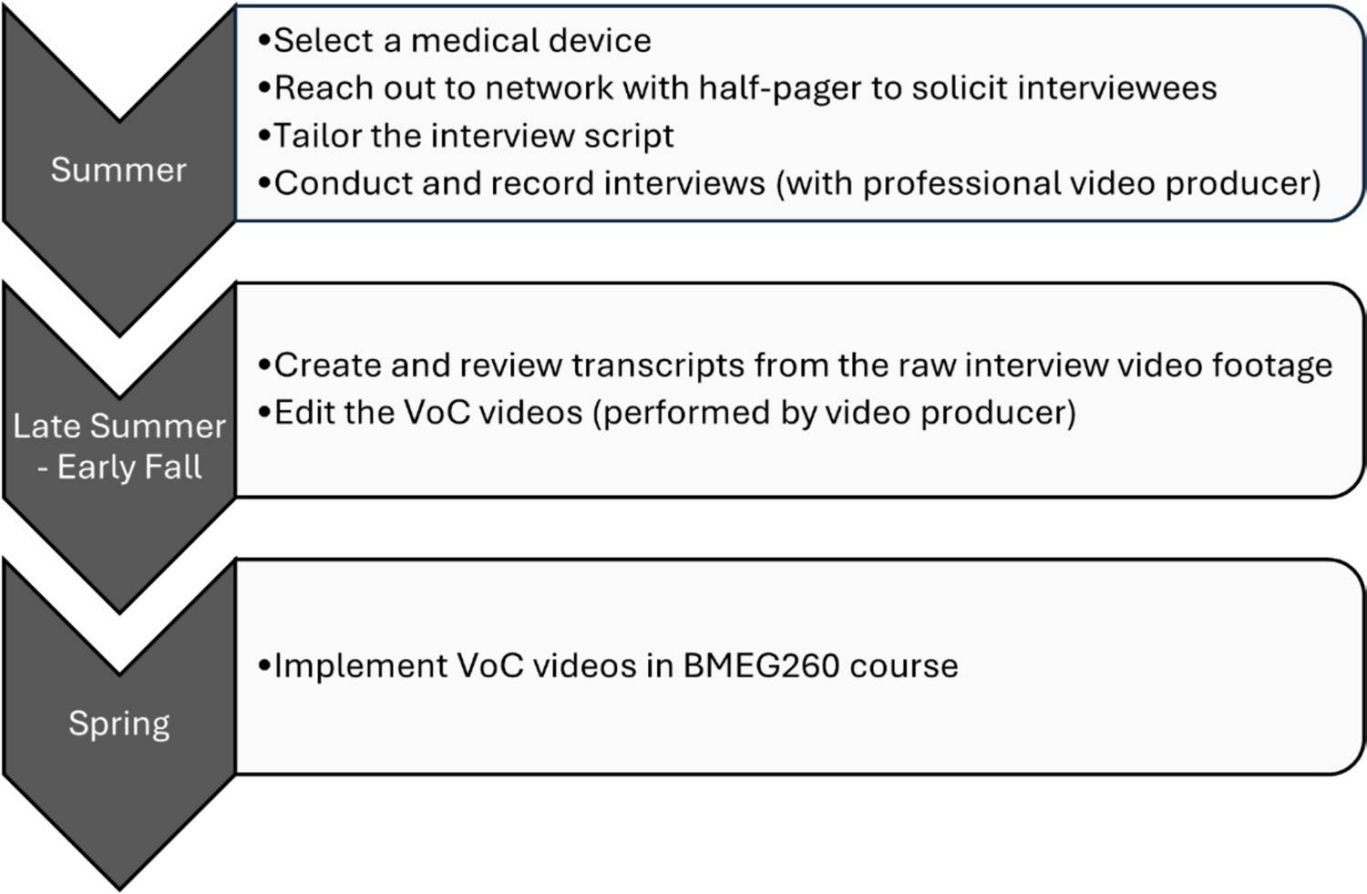
Process used to create VoC videos. Creation of the VoC videos started in the summer, with most of the interviews taking place before the fall semester began. All videos were edited by the end of the fall semester for subsequent use in the spring semester. This process was repeated for a total of 4 cycles to create a collection of videos focused on 6 different medical devices

Creation of the full suite of VoC videos occurred over four annual cycles, with a different medical device focus each year, dependent on the needs of the course in which the videos would be used. The process we followed each year to create a new set of medical device videos is depicted in Fig. 1.

To start, the lead author/course instructor wrote a half-page document that summarized the project and asked medical device users for participation in an interview. This document was emailed to relevant individuals in her network. Potential participants were informed in advance to expect a 30-minute, video-recorded interview focused on their experiences with the medical device, including how/why they use it or recommend its use, what features work well, and what challenges they have experienced. The interview script was shared with those who chose to participate. Sometimes additional permissions or public relations (PR) escorts were required for on-site filming. All VoC interviewees volunteered their time and were not paid.

A semi-structured interview approach was used for all interviews. A generalized set of questions is provided in Online Appendix A. These questions were tailored for each of the six medical devices and for the different types of stakeholders being interviewed. Open-ended questions were prioritized, and questions considered images (descriptions of using the product), past issues, current considerations, and future enhancements, following the practices described by Burchill and Brodie for conducting VoC interviews [28].

The lead author and professional video producers made some deliberate decisions when creating and editing these VoC videos, as described in Table 1. One key decision was to focus on a single interview for each video rather than “montages” that integrate multiple interviews. Although montages may be more interesting to watch and have a more focused message, they would reduce the purity of the interviewee’s telling of their experience and would inherently introduce bias. Important to this decision is the fact that these VoC videos are meant to be educational tools for learners to develop skills in needs finding and development of design criteria. For example, a montage of clips where multiple interviewees all describe a similar challenge with a medical device would be sourcing the unmet need, thereby doing the work for the students and reducing the intended educational purpose of the video. Furthermore, we wanted students to focus their cognitive effort on identifying challenges in using medical devices—not on trying to follow the interviewee’s verbal delivery. To support this, we rearranged the questions and responses into a logical sequence, aiming to balance the authenticity of the interview with its educational purpose and the clarity of the final videos.

**Table 1:** Decisions made when filming and editing the VoC videos

Decision	Rationale
Prioritize in-person interviews	Attain higher-quality footage compared to a virtual interview
During filming, focus only on the person being interviewed	Ensure that the interviewee’s experience is central to the viewer
Limit the interview to 30 minutes (typically)	Respect the time of the people being interviewed Keep the final, produced video to a reasonable length for viewers to watch
Each video is an individual interview with limited edits to content to retain most of the original interview	Minimize biasing the content
Rearrange questions/responses	Create a logical sequence for the viewers
Incorporate B-roll only when possible and meaningful	Create visual interest in the video Keep the focus on the interviewee

### Implementing VoC Videos in a Course

These videos have been implemented in a required, sophomore-level course, BMEG260 Introduction to Medical Device Design. This course has been summarized previously [29]. In brief, throughout the semester, student teams learn about three different medical devices. At the end of each medical device unit, teams submit multiple industry-based deliverables, using provided templates that address both how the current devices work and what could be done to improve them. The VoC videos for each medical device unit are posted to the course learning management system. The students are told that they should start each new unit by watching all the posted VoC videos. Student teams are expected (explicitly stated in the assignment instructions) to use the VoC videos to inform their development of a need statement and design inputs for a future, improved device. In separate lessons early in the semester, students are introduced to need statements, following the template described in the *Biodesign* textbook [30], and design inputs. For design inputs, the lesson starts with an explanation of how the voice of the customer leads to functional requirements, which leads to design requirements. This lesson introduces the “waterfall” diagram and traceability matrix and provides examples (unrelated to the devices covered in the class) of design inputs that were informed by VoC input. In both lessons, the instructional emphasis has been on the importance of incorporating VoC and translating it into useful information, rather than on how to “capture” it or on how to perform “synthesis/analysis.”

Student teams are asked to justify the problem, population, and outcome defined in their need statement by including the Voice of the Customer and additional references. Their need statement may be focused on either an incremental improvement to existing devices or on a new need with no available solution. The design inputs (functional and design requirements) are expected to align with the need statement. At least some of the design inputs must reflect the Voice of the Customer, which is articulated by pasting direct quotes from the VoC videos into their design inputs table, acting as sources for the requirement. Students were provided with rubrics in advance that included evaluation criteria on their use of VoC in their need statements and design inputs.

### Assessments

Assessments used to evaluate creation of the VoC videos, scalability, the three research questions, and overall student perspectives of the VoC videos are summarized in Table 2. The evaluation protocol was submitted to and granted exempt status by the University of Delaware IRB.

**Table 2:** Assessment matrix

Item assessed	Assessment strategy
Creation of VoC videos for different medical devices	- Number of videos created for each medical device - Average video length
Scalability of VoC videos	- Number of years videos have been used in BMEG260 - Number of students enrolled in the course
RQ1: Are students able to use VoC videos to inform their development of need statements and design inputs?	- Review team deliverables for whether VoC videos were referenced for the need statement and design inputs (spring 2025) - Pre- and post-course survey using one subdimension from the Engineering Design Self-Efficacy Instrument [31] (spring 2024, spring 2025) - Post-course survey question (spring 2025) - Focus groups (spring 2024, spring 2025)
RQ2: Do recorded VoC videos help students develop empathy for users of medical devices?	- Pre- and post-course survey using an adaptation of the Empathy in Design Scale EMPA-D [32] (spring 2025) - Post-course survey question (spring 2025) - Focus groups (spring 2024, spring 2025)
RQ3: Do recorded VoC videos foster engagement in course content?	- Post-course survey question (spring 2025) - Focus groups (spring 2024, spring 2025)
Student perspectives of VoC videos	- Focus groups (spring 2024, spring 2025)

#### Student Team Deliverables

To directly assess whether students were able to use the VoC videos to inform their development of need statements and design inputs, the team deliverables (need statements and design inputs) from all three medical device units in spring 2025 were scored by a single evaluator using a 0–4 scale (refer to Online Appendix B for rubrics used). These rubrics are similar to the ones that were used for grading submissions in the class. For this study, the quality of the need statements was not assessed because multiple factors can influence the quality beyond the application of the VoC videos. Instead, we focused on whether students were able to reference the VoC videos in their need statement justifications and design inputs tables. Descriptive statistics are reported. Comparative statistics (between units/over time) are not reported because they do not align with the research questions.

#### Surveys

Surveys were used to assess all three research questions. All students enrolled in BMEG260 were asked to complete a survey at the beginning (i.e., pre-survey) and again at the end of the semester (i.e., post-survey). Students received an email from a professional external evaluator containing a URL for the online survey. The consent page of the survey stated that participation was voluntary and no identifiable information is collected. Students could elect to consent (“I agree”) or not (“I disagree”). The instructor set aside time to complete the survey at the start of the first (pre) and last (post) classes. Multiple follow-up emails were sent to increase the response rate. The survey covered many different topics, as it is part of a long-term project with multiple aims, but here we will describe and report on only those questions relevant to the VoC videos.

The pre- and post-course survey in spring 2024 and 2025 included Carberry’s validated Engineering Design Self-Efficacy Instrument [31]. In this instrument, respondents rate nine aspects (subdimensions) of engineering design for confidence, motivation, ability to succeed, and anxiety using a 0-100 scale. The subdimension of engineering design on this instrument that aligns most closely with the purpose of the VoC videos is “Identify a design need.” In this paper we report only on the findings from this subdimension of engineering design, as noted in Online Appendix C. Responses from both years were considered independently. As a Shapiro–Wilk test indicated that the data were normally distributed, paired t-tests were used to examine score changes from pre-survey to post-survey, performed using the Statistical Package for the Social Sciences (SPSS) version 31. Only responses from students who completed both the pre- and the post-survey are reported. Statistical comparisons between cohorts (2024 vs. 2025) are not reported because they do not align with the research questions.

We added questions to the 2025 iteration of the survey to reflect that our research questions became more refined over time, and to include an adaptation of a newly published survey instrument that aligned with our research questions. The pre- and post-course survey implemented in spring 2025 included questions adapted from the Empathy in Design Scale (EMPA-D) [32]; these questions were not included in the 2024 survey. In this validated, 11-item instrument, respondents are asked to rate on a 1–7 Likert-based scale how well each statement describes them. The scale produces a total empathy score and sub-scores for three dimensions: emotional interest, personal experience, and self-awareness. To adapt the questions to our needs, we modified “employee” to “Biomedical Engineering student” or “Biomedical Engineer” and “services” to “medical devices.” The adapted survey questions are provided in Online Appendix C. As a Shapiro-Wilk test indicated that the data were normally distributed, pre-post-statistical analysis was conducted via paired *t* tests to determine changes in empathy scores. Only responses from students who completed both the pre- and the post-survey are reported.

In addition to the pre–post questions, we also asked in spring 2025 one end-of-semester question specific to each research question (RQ1-3). These questions are provided in Online Appendix C. Respondents used a 1–5 Likert-based scale to rate each question, with an additional option for students to respond that they did not watch the videos. Descriptive statistics for these three questions are reported.

#### Focus Groups

Focus groups were used to assess all three research questions and to collect overall student perspectives of the VoC videos. The in-person focus groups were led by a professional external evaluator at the end of the spring 2024 and 2025 semesters during a studio class period, for a total of three discussions in each year. The instructor was not present. Discussions were audio-recorded with the students’ permission, transcribed by a professional transcription service, coded, and examined using thematic analysis. The focus groups covered many aspects of the course; however, here we report only on the questions that relate to the VoC videos, as provided in Online Appendix D. Example student quotes that support the identified themes are provided.

## Results

In total, 37 interviews were captured, as summarized in Table 3. 35 of the final VoC videos are available online at https://sites.udel.edu/meddevicesvoc/. Edited videos ranged from 7 to 43 minutes in duration, with an average length of 23 minutes. Although the interviews were intended to last 30 minutes, six interviews went longer, and it felt important to include what the interviewees said. These videos have been used successfully across 4 years of offering the BMEG260 course (1 pilot year + 3 full offerings), reaching 157 students and demonstrating success in scalability.

**Table 3:** Summary of completed video interviews

Medical device	Video interviews	Year recorded	Years used in BMEG260
Surgical Staplers	5 total 4 surgeons (pediatric, oncology, thoracic, bariatric) 1 surgical technologist	2021	2022, 2024, 2025
Breast Pumps	5 total 2 OB-GYN physicians & former breast pump users 1 International Board-Certified Lactation Consultant (IBCLC) 1 speech language pathologist, lactation counselor, orofacial myofunctional therapist, & former breast pump user 1 former breast pump user (no clinical training)	2021	2022, 2023, 2024
Stents	4 total 2 interventional cardiologists/vascular surgeons 1 cardiac surgeon 1 structural interventional cardiology fellow	2021	2022, 2023, 2025
Wearable EMG Biofeedback	5 total 5 physical therapists	2022	2023
Cochlear Implants	9 total 2 cochlear implant users 3 otolaryngologists 3 audiologists 1 MD-PhD academic researcher/innovator	2023	2024
Continuous Glucose Monitors (CGM)	9 total 5 CGM users (1 is a caregiver to a CGM user) 1 pediatric endocrinologist 1 co-founder/president of a company that uses CGMs 1 PhD academic researcher/engineer 1 PhD academic/clinical researcher/nurse	2024	2025

### Student Team Deliverables

For each of the three medical device units in spring 2025, 15–16 teams submitted need statement and design input deliverables. Unit 1 was surgical staplers, unit 2 was continuous glucose monitors, and unit 3 was stents.

**Fig. 2:**
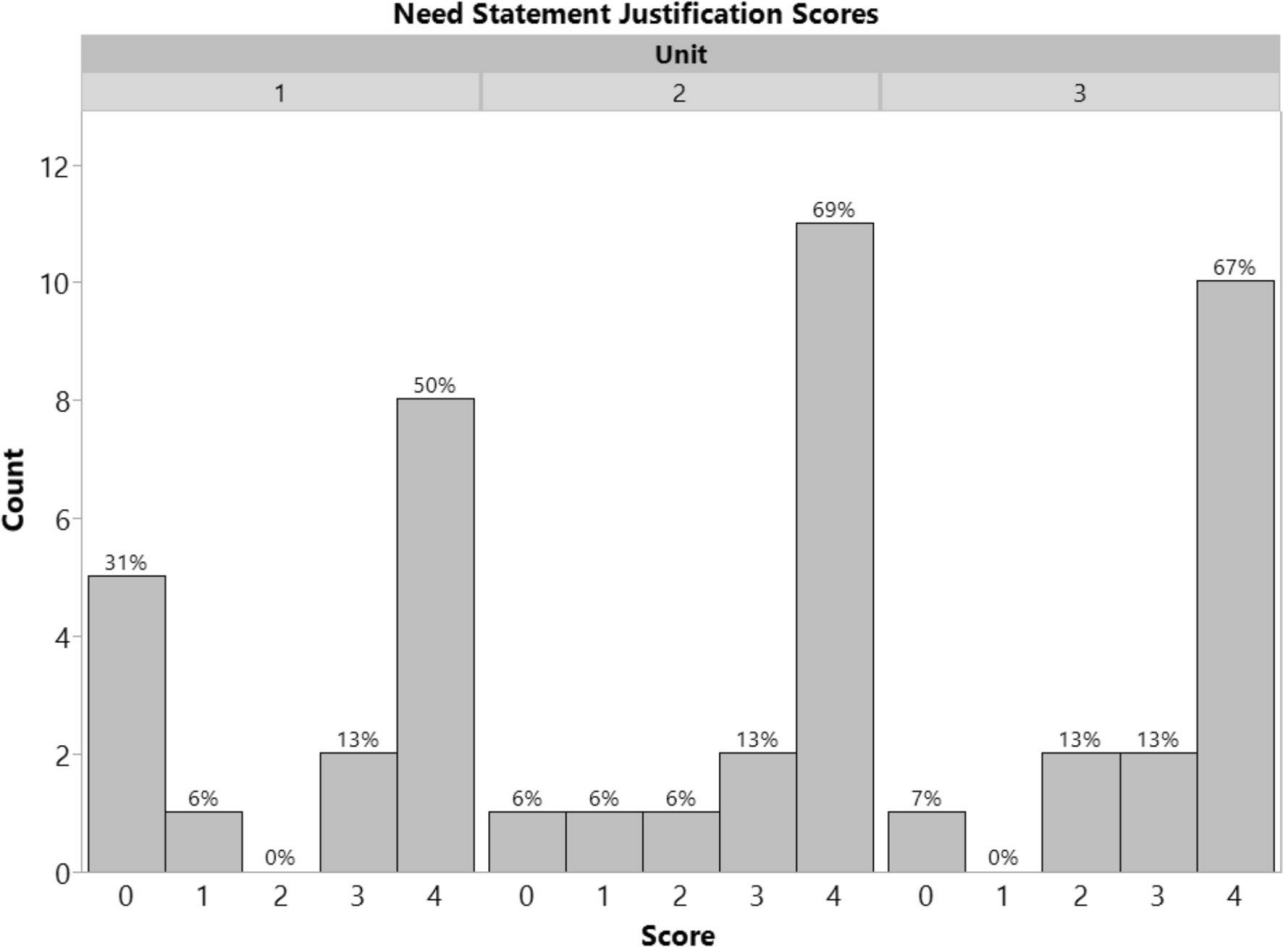
Histogram of scores (0–4 scale) assessing the extent to which VoC videos were used as justifications for need statements. Student teams were able to effectively apply the VoC videos to inform the development of their need statements

In total, 47 student team submissions were evaluated for their ability to incorporate the VoC videos as justifications for their need statement. A histogram of scores is shown in Fig. 2. Descriptive statistics using a 0–4 scale are reported in Table 4. Overall, 63% of submissions in unit 1, 82% of submissions in unit 2, and 80% of submissions in unit 3 demonstrated proficiency in applying the VoC videos to need statement development. Two examples of student work that scored a 4 on applying VoC to justify their need statements are provided in Online Appendix E.

**Table 4:** VoC videos in need statement justifications-student team deliverable scores (0–4 scale)

	Unit 1	Unit 2	Unit 3
Mean ± StDev	2.4 ± 1.9	3.3 ± 1.3	3.3 ± 1.2
Median	3.5	4	4
N	16	16	15

**Fig. 3:**
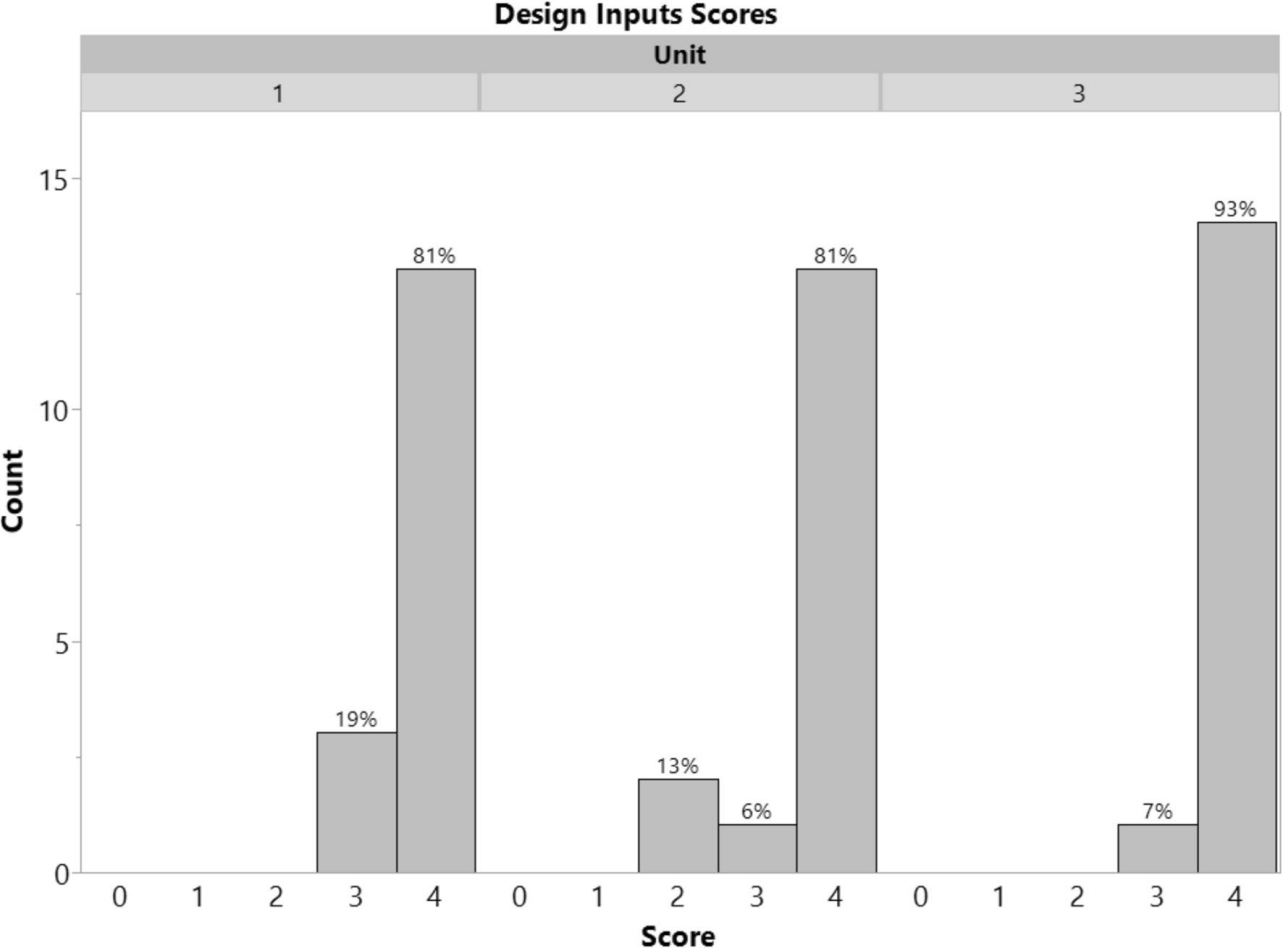
Histogram of scores (0–4 scale) assessing the extent to which VoC videos were used as sources for design inputs. No teams scored 0 or 1. Student teams were able to effectively apply the VoC videos to inform their design inputs

In total, 47 student team submissions were evaluated for their ability to incorporate the VoC videos as sources for their design inputs. A histogram of scores is shown in Fig. 3. Descriptive statistics using a 0–4 scale are reported in Table 5. Overall, 100% of submissions in unit 1, 87% of submissions in unit 2, and 100% of submissions in unit 3 demonstrated proficiency in applying the VoC videos to design inputs development. Two examples of student work that scored a 4 on applying the VoC videos as sources for their design inputs are provided in Online Appendix E.

**Table 5:** VoC videos as design input sources-student team deliverable scores (0–4 scale)

	Unit 1	Unit 2	Unit 3
Mean ± StDev	3.8 ± 0.4	3.7 ± 0.7	3.9 ± 0.3
Median	4	4	4
N	16	16	15

### Surveys

**Fig. 4:**
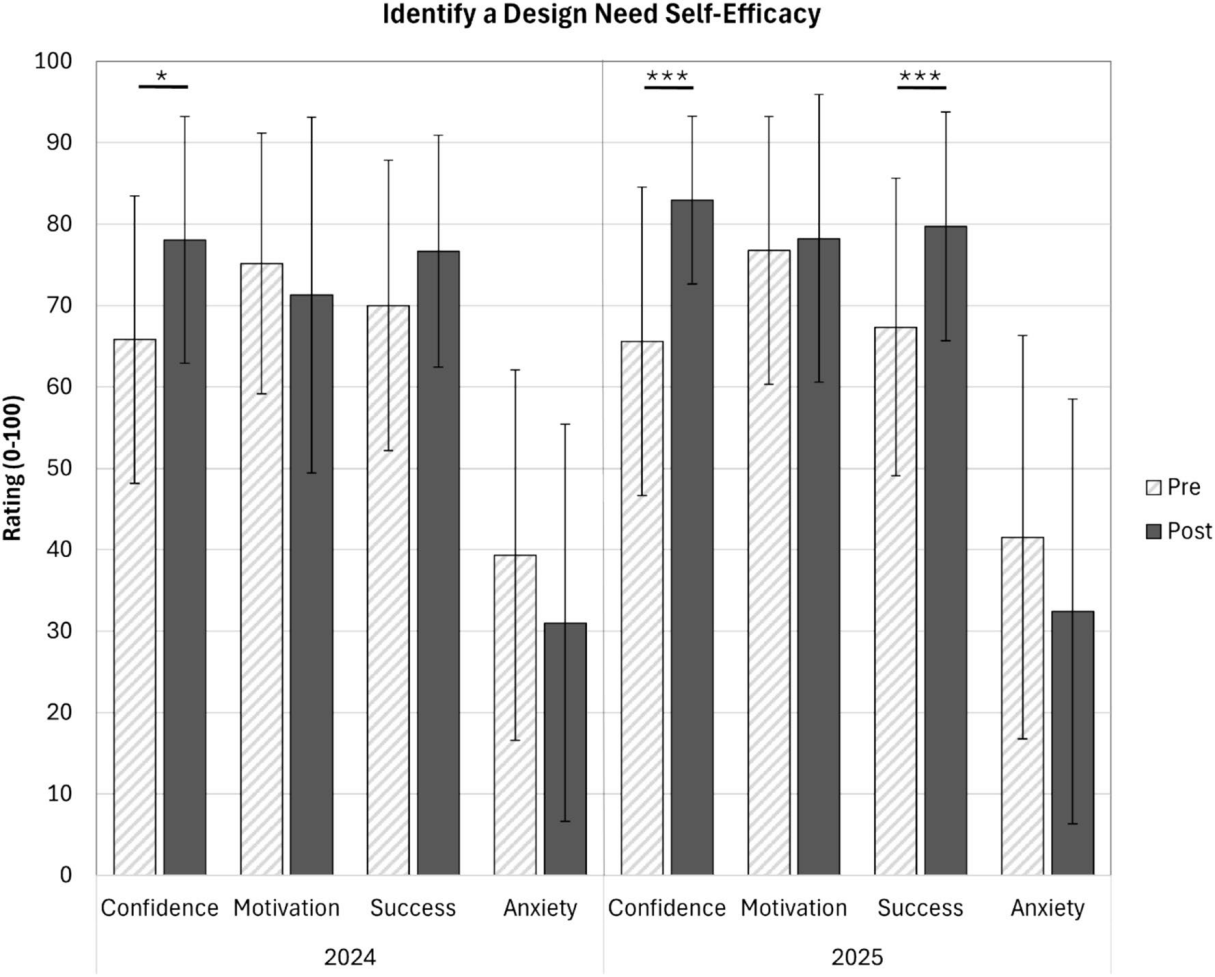
Post-course ratings for students’ confidence in their ability to identify a design need increased significantly compared to pre-course in 2024 and 2025. A statistically significant increase in students’ ratings for their expected success in identifying a design need was also detected in the 2025 cohort. (bars = mean ± standard deviation, pre = light hashed bars, post = dark solid bars, *p < 0.05, ***p < 0.001, 2024 *N* = 33, 2025 *N* = 29–34)

Using Carberry’s Engineering Design Self-Efficacy Instrument [31], students demonstrated increased confidence in their ability to identify a design need at the end of the semester compared to the start of the semester (Fig. 4). This increase was detected in both 2024 (*p* < 0.05) and 2025 (*p* < 0.001) offerings of the course. Additionally, students demonstrated increased outcome expectancy (perceived likelihood to succeed) in 2025 (*p* < 0.001). There were no statistically significant changes for motivation or anxiety in either year, although anxiety decreased in both years.

**Fig. 5:**
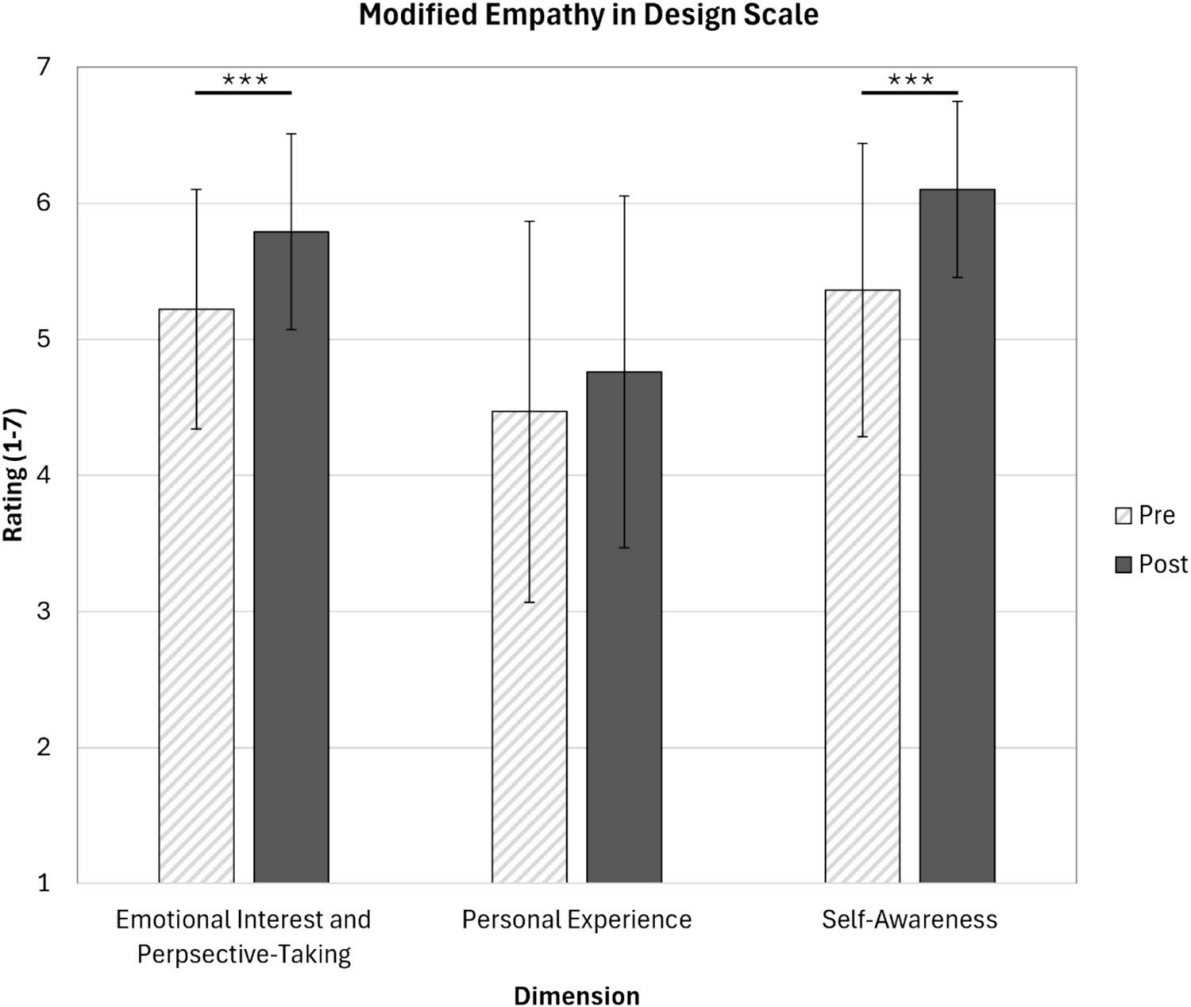
Post-course student ratings for the dimensions of Emotional interest and perspective taking and self-awareness on a modified version of the EMPA-D scale increased significantly compared to pre-course. (bars = mean ± standard deviation, pre = light hashed bars, post = dark solid bars, ***p < 0.001, *N* = 33–34)

Using a modified version of the Empathy in Design Scale [32], the Emotional Interest and Perspective Taking dimension moderately increased from 5.22 to 5.79 (*p* < 0.001, Fig. 5), indicating an enhanced student ability to consider and emotionally relate to medical device users’ experiences. The Self-Awareness dimension exhibited a gain from 5.36 to 6.10 (*p* < 0.001, Fig. 5), suggesting that students became more attuned to their own emotional responses and how these affect their interactions with medical device users. Moreover, although students’ empathy, as it relates to the dimension of Personal Experience, saw a slight increase (from 4.47 to 4.76, Fig. 5), the change was not statistically significant. Additionally, 10 of the 11 individual items on the scale statistically increased post compared to pre (Online Appendix F).

**Fig. 6:**
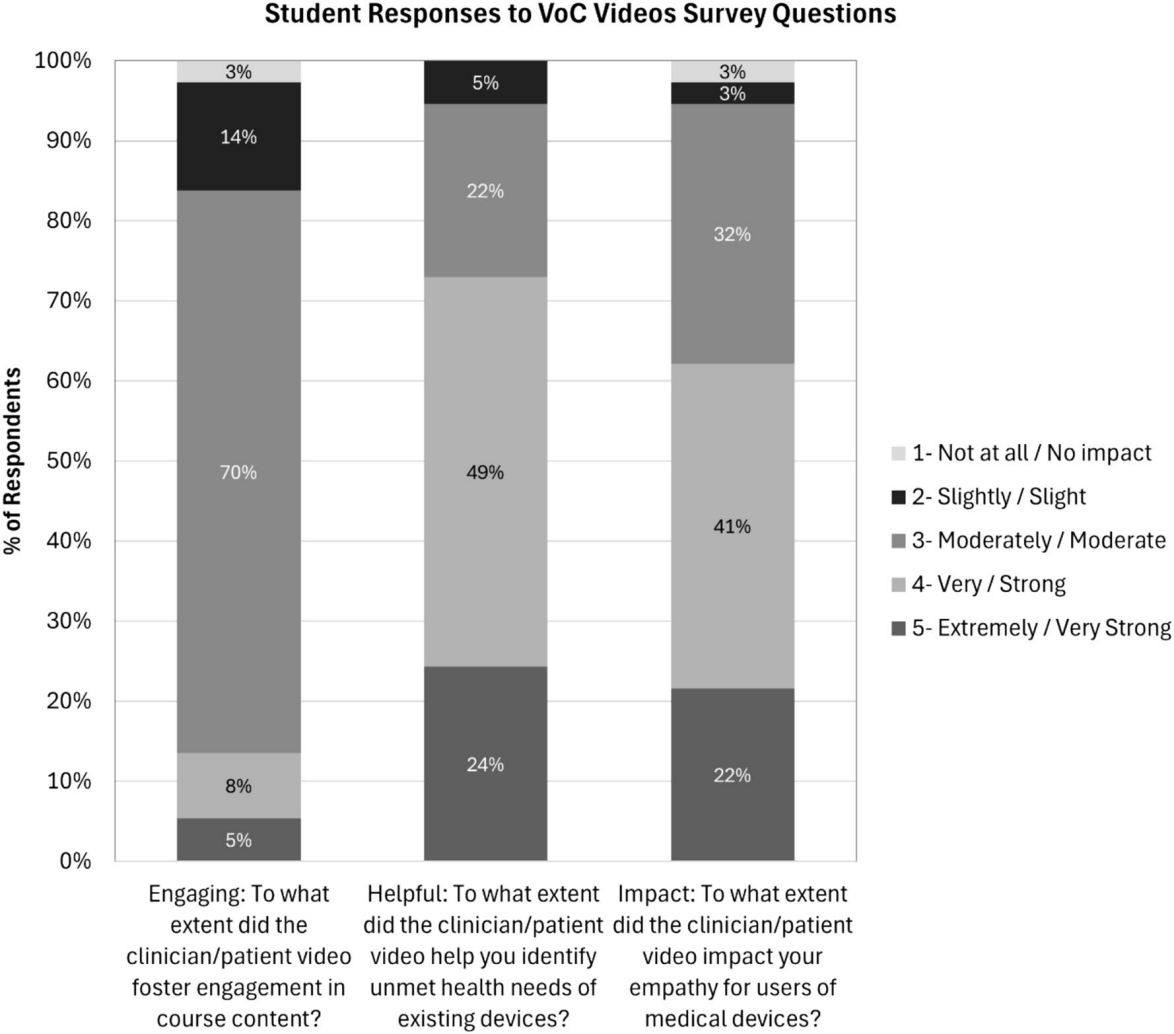
Most student respondents on an end-of-semester survey reported that the VoC videos fostered engagement in course content, helped them identify unmet needs, and impacted their empathy for users of medical devices. No respondents reported that they did not watch the VoC videos. (*N* = 37)

Three questions were asked in an end-of-semester survey to address the primary research questions. No students selected the option “I did not watch the clinician/patient videos.” Of the 37 responses, most students (31/37, 84%) felt that the VoC videos at least moderately enhanced their engagement in course content (Fig. 6, mean = 3.0/5, median = 3/5). 95% (35/37) of students reported that the VoC videos were at least moderately helpful in identifying unmet needs of existing medical devices, with the majority reporting the VoC videos to be extremely or very helpful (Fig. 6, mean = 3.9/5, median = 4/5). Lastly, 95% (35/37) of students felt that the VoC videos had at least a moderate impact on their empathy for users of medical devices (Fig. 6, mean = 3.8/5, median = 4/5).

### Focus Groups

Based on focus groups conducted with the 2024 and 2025 cohorts, students generally appreciated the comprehensive and human-centered insight offered by the VoC videos. In particular, they found value in hearing from different stakeholders and diverse perspectives. Moreover, they found the VoC videos complemented traditional academic sources and facilitated the identification of real-world problems in design. Additionally, the VoC videos helped students build empathy by exposing them to the emotional realities of patients, particularly vulnerable users like children, and by surfacing design considerations tied to esthetics, stigma, and long-term burden. Despite these findings, one consistent theme that emerged from discussions with the students in the 2025 (but not 2024) cohort is that the videos and their transcripts were too long or at times redundant. Example student quotes supporting these themes are presented in Table 6.

**Table 6:** Focus group themes and example student quotes

Focus group theme	Example student quotes
Students valued hearing from different stakeholders and diverse perspectives through the VoC videos	“A lot of the stuff that you’re doing, it’s literature searches and especially with this last unit, it really makes you think of different perspectives. So I am not someone who would use a cochlear implant […]. And so initially I had thought, “Oh, they hear just the way I do.” But it’s different and so their needs are going to be different based off of that. So I think that was very helpful, just having a way to have different perspectives that was really helpful.” (2024) “Yeah, because you heard it from... It wasn’t just doctors and clinicians. Sometimes, you got to hear the perspective of the patient. […] The video is for a patient’s family member, […] an engineer, so you got to hear multiple perspectives on a problem. And sometimes that gave me the creativity to I guess work through especially the first form where you have to come up with a problem statement. I think those videos were helpful for that.” (2025) “I like the different types of videos. […] trying to figure out what we wanted our new device to be better than the ones that we’re referencing, it was nice that there was different perspectives and so there’s different things that you could try to tackle. I know for the so-called stapler, we sort of took our main idea that we wanted to tackle from a very specific video. There’s other videos that had other issues, so it made that you’re not sort of funneled into fixing the one problem and that you could sort of branch out and figure out on your own what you’re more comfortable doing.” (2025)
VoC videos complemented traditional academic sources and facilitated the identification of real-world problems in design	“You’re reading about these devices from journals and stuff like that, but actually hearing the surgeons talk about the surgical staplers they’re using and what’s good about it, what’s not good about it and how that differs from person to person and then actually hearing from the people using the other devices like the breast pumps and the cochlear implants, I feel like that’s hearing it from more of a not scientific engineer point of view. That’s the real people talking about what it’s like for them and how it affects their life. So, I think that gives an extra... It gives a fuller picture, I think, of the problems and the good things too so I thought those were really, really helpful.” (2024) “I really liked it. It was real. It wasn’t like, “Oh, go Google and see what the problems are.” We actually got real people talking about it and […] it wasn’t just the person who was affected by it was everybody around them too. When we were learning about cochlear implants and stuff, I didn’t even think to think about the speech pathologist. That wasn’t someone where I was like, oh, that’s definitely involved because I didn’t know the process well enough. So when I was hearing about that from the VoC that was nice to hear because that’s not something you always think about. And if you told us to go search for problems, I don’t think anyone would look up the time it takes a speech pathologist to help or like that.” (2024) “I liked how she brought in a broad range of perspectives. For example, for the breast pumps, there were doctors and then there were users and then there were doctors that were also users. So, there was just a lot of point of views that you could talk from and I feel like that’s the most important thing is from the customer. […] Hearing it from the customer is really important and it’s also, I thought that the videos were nice because sometimes it’s hard to research that stuff online, so it’s better to just hear it from a direct person rather than skimming through papers and statistics and stuff.” (2024)
VoC videos helped students build empathy	“I would definitely agree with the empathy factor, particularly for the breast pumps because there were some people who... I mean, with breast pumps, you’re talking about feeding a child so I think inherently it’s a little bit more vulnerable of a subject and just hearing people explain how they had problems with their baby and how that affected their mental health and the baby’s well-being, I think it just kind of gave me at least a better understanding of the issue at hand and why it’s important and how much it affects people.” (2024) “Yeah, I mean you definitely feel the empathy when you’re hearing and listening and watching them react to it rather than reading a document that says, oh, this screwed up for this reason back in this year. And it was also really interesting to learn how it affected them, but also what they’ve needed to do to compensate that or adjust to life with the issue or something. So that just really made it more, it humanized the problem rather than just reading it on a piece of paper.” (2024) “I’ll just say like, the glucose monitors, one of the voice of customers was a little girl. And I felt a lot of empathy for her because she was, like, “Oh, I just want it to look cute,” and things like that, so things like maybe an engineer wouldn’t typically think about, “Oh, a child could be wearing this. We should have stickers put on them or make it a fun color.” Or I know someone was, like, “Oh there’s stigma around diabetes and things like that, and I don’t like it being that visible because people could perceive me in some way.” So I feel like in that way it helped me, things that I feel like an engineer wouldn’t think about. They think about the function, it doing what it’s supposed to do, but also thinking about, I guess, more like the societal impacts and how the person feels about it.” (2025)
VoC videos and their transcripts were too long	“I just thought they were so long. And I ended up reading the transcripts more than watching the video. I thought that was very helpful, but, even then, the transcripts were long. And there was multiple of each of the videos every time, so it was just so much information I feel like thrown at you. And it was helpful information, but a lot of them had the same point I feel like. If I’m being honest, I kind of would just take the transcript, put it in ChatGPT and summarize them.” (2025) “Yeah. I mean not too long because... I mean they had a lot to say, and it was all very important, but I think, in the grand scheme of things, it was a little overwhelming.” (2025)

### Results Summary

In the sections above, we have presented results from surveys, focus groups, and direct assessments, in addition to demonstrating scalability. These results are summarized in Table 7.

**Table 7:** Summarized results show the benefits of the VoC videos

Item assessed	Summarized results
Creation of VoC videos for different medical devices	- 37 videos total (4–9 per device for 6 devices) - average duration 23 minutes (range: 7 to 43 minutes)
Scalability of VoC videos	- used for 4 years of offering BMEG260 - 157 students enrolled in the course over 4 years - ongoing use in BMEG260 - videos publicly available: https://sites.udel.edu/meddevicesvoc/
RQ1: Are students able to use VoC videos to inform their development of need statements and design inputs?	- 63–82% of student team submissions appropriately referenced VoC videos in need statement justifications - 87–100% of student team submissions appropriately used VoC videos as sources for design inputs - increased student confidence and expected success in identifying a design need based on the Engineering Design Self-Efficacy Instrument [31] (*p* < 0.05) - 100% of students found the VoC videos at least slightly helpful in identifying unmet needs - Focus group discussions supported that students used the VoC videos to develop their need statements
RQ2: Do recorded VoC videos help students develop empathy for users of medical devices?	- increased student empathy for users based on an adaptation of the Empathy in Design Scale EMPA-D [32] (*p* < 0.05) - 97% of students reported that the VoC videos impacted (at least slightly) their empathy for users of medical devices - focus group discussions supported that students developed empathy through learning about different medical device users’ perspectives
RQ3: Do recorded VoC videos foster engagement in course content?	- 97% of students reported the VoC videos were at least slightly engaging - although focus groups identified that students found the VoC videos helpful, the 2025 cohort felt they were too long and sometimes redundant

## Discussion

Our collection of VoC videos consists of targeted and specific interviews of medical device users to bring the voice of the customer to the student. In the VoC videos, stakeholders narrate their personal experiences with the specific medical device. By presenting multiple (4–9) user interviews per medical device, we have captured different perspectives on the same topic.

Demonstrating successful scalability, the VoC videos have been used across multiple years in our undergraduate biomedical engineering program. Overall, clinical immersion programs average an enrollment of 27 students per year [5]. We are able to reach the entire cohort of sophomore-level biomedical engineering students (typically 45–70 students annually). In contrast to the national average of 36% of students participating in clinical immersion [5], 100% of our undergraduate biomedical engineering student population will benefit from exposure to needs-finding skills through the VoC videos.

### RQ1: Are Students Able to Use VoC Videos to Inform Their Development of Need Statements and Design Inputs?

Through directly evaluating team submissions across three different medical device units, we found that student teams were able to effectively use the VoC videos to inform their development of need statements and design inputs. Nearly all teams demonstrated proficiency. Teams that received a score of 0 for applying the VoC videos to their need statements typically failed to include a justification or direct reference to the videos. Although their need statements typically aligned with the experiences described in the VoC videos, this alignment alone, without explicit evidence of having engaged with the VoC videos—such as citations or quotes—was insufficient. Thus, even when alignment was apparent to the instructor, teams were scored based on the absence of documented application rather than inferred understanding. This finding suggests that clearer instruction on how to write strong justifications and reference the VoC videos is warranted. This conclusion is further supported by the finding that unit 1 had the lowest need statement justification scores, which is more of a reflection of students misunderstanding the expectations rather than a reflection of the efficacy of the VoC videos. Scores were higher in units 2 and 3 compared to unit 1, likely because students had received feedback that they need to directly reference the VoC videos to justify their need statements.

Furthermore, the number of VoC videos watched and used by each team for each unit is unknown. It was commonly observed that students would use just one quote from one VoC video as justification, which contrasts the focus group finding that the VoC videos sometimes had redundant information; therefore, better instruction on the importance of identifying needs from multiple sources (i.e., deliberate instruction on the “synthesis/analysis” stage of needs exploration) is an area to improve moving forward. More specifically, the “Requirements Path,” method of analyzing VoC data, as described by Burchill and Brodie [28] and implemented in medical device development [2], could be a useful model for instruction.

This study did not include direct assessment of the quality of the need statements generated by the students. Because the study lacks a control group, conclusions about the quality as it relates to applying the VoC videos cannot be made; however, investigating need statement quality by implementing strategies described by others [33, 34], may be a future area of interest. In addition, all unit deliverables were submitted as a team, so individual abilities to apply the VoC videos to develop need statements and design inputs were not assessed. Lastly, we observed that most need statements centered around improvements to existing devices (e.g., a better way to attach continuous glucose monitors to users), but some student teams did focus on “new” needs (e.g., a way to measure a variety of other hormones that influence glucose levels).

Further supporting the effectiveness of the VoC videos in helping students identify unmet needs, student confidence and perceived likelihood to succeed in their ability to identify a design need increased after taking the course. Through focus group discussions, students commented on being able to use the videos to develop their need statements. Lastly, 73% of student survey respondents found the VoC videos very or extremely helpful in identifying unmet health needs of existing devices. Taken together, the direct assessments, survey results, and focus group findings indicate that students are able to use the VoC videos to develop need statements and design inputs.

### RQ2: Do Recorded VoC Videos Help Students Develop Empathy for Users of Medical Devices?

Through adapting a validated survey instrument focused on empathy toward users, we measured statistically significant increases in the dimensions of Emotional Interest and Perspective Taking and in Self-Awareness at the end of the course compared to the start. Emotional Interest and Perspective Taking measures the students’ willingness to understand and consider the emotional state and perspective of others [32]. Self-Awareness measures students’ conscious understanding of their own feelings and how they might impact their ability to empathize with others [32]. Although increased, there was no statistically significant difference in the dimension of Personal Experience, which measures how much an individual draws upon their own past experiences to understand and relate to the emotions of others [32]. The finding of no change in this dimension makes sense, as this course was not focused on students directly experiencing these medical conditions or devices (although students did have some interactions with the devices).

On the post-course survey, 62% of students reported that the VoC videos had a strong or very strong impact on their empathy for users of medical devices, with 97% reporting that the VoC videos had at least a slight impact. Focus group discussions lend further support with several comments expanding upon this theme.

By nature of the study design and the fact that the course is required of all students in our program, there is no control group. Instead, pre- and post-course assessments were implemented to allow for paired comparisons, which strengthen the conclusions we can draw. We believe the changes in empathy that we measured are most heavily influenced by the VoC videos; however, we cannot rule out other influences from within or outside of the course.

Within this course, there are three other elements that we think could contribute to empathy development. First, we bring in guest speakers, which has been a notable highlight of the course for the students. Most of these guest speakers work in the medical devices industry. Sometimes, these speakers emphasize the importance of capturing VoC to inform medical device design; however, they are speaking as the engineers and sales representatives working with the devices, not as the users themselves. The statements in the modified empathy in design scale center around users’ experiences. Secondly, the students have some interactions with the devices themselves. For example, they learned how to fire surgical staplers, had the option of using an over-the-counter continuous glucose monitor if they chose, and were able to see and feel stents. Through their own experiences with these devices, the students may be developing empathy for the device users; however, if these device interactions were a significant contributor to empathy development, then we would expect the “Personal Experience” dimension of the modified empathy in design scale to demonstrate greater gains. Instead, this dimension was the only one that did not achieve statistical significance. Lastly, students may develop empathy through other resources (articles that they read, other videos that they find online, etc.); however, one of the emergent themes of the focus groups was that the students valued how the VoC videos complemented traditional academic sources and “humanized” the problems for them. The focus group results and the end-of-semester question (where 97% of respondents indicated at least slight impact on empathy) both center on the impact of the VoC videos specifically in developing empathy, and they lend strong support to the quantitative findings from the pre-post modified empathy in design scale. Taken together, we believe this evidence supports that the VoC videos have helped students develop empathy for users of medical devices.

### RQ3: Do Recorded VoC Videos Foster Engagement in Course Content?

In this study, students most frequently reported that the VoC videos moderately fostered their engagement with course content (70%), with 97% reporting that the VoC videos at least slightly fostered engagement. Focus groups supported this notion, particularly when the VoC videos were paired with the students’ own hands-on experiences with the devices. However, an emergent theme from focus groups with the 2025 cohort was that the videos were too long or sometimes redundant, and students would look for shortcuts to complete the necessary work. The ideal length of the VoC videos to ensure that they are engaging and a reasonable time commitment for the students to watch, while capturing the experiences of the users and serving as an educational tool (instead of feeding the synthesized insights directly to the students) is still unknown. Combining the interviews with recordings of using the medical devices in their normal environments, adding an “observation” element, may produce a more visually interesting video to engage the viewers; however, this may further increase the video length, would be logistically challenging to execute, and may be unnecessary because many procedure videos are already available online.

“Engagement” is a complex phenomenon that is often considered in cognitive, behavioral, and emotional dimensions [35]. This study did not distinguish these components of engagement, though we suspect that there is an emotional engagement element to the VoC videos, given the measured changes in and student self-reports of empathy for users of medical devices. Since the students were also required to use the VoC videos as support for their need statement and design input generation, this lends itself to engagement in both the cognitive and behavioral domains.

This study is limited by a lack of direct assessments of student engagement with the VoC videos, such as quantifying video views from all students, using eye tracking and pupil dilation [36], or measuring heart rate, electrodermal activity, skin temperature, or body movement [37]. The students were required to reference the VoC videos in their deliverables for each medical device unit in the course; however, because deliverables were submitted as a team, it is unlikely that all students watched all VoC videos. Despite this, the students were also required to rotate roles on their teams throughout the semester, so we expect that all students should have watched at least some of the VoC videos. This is supported by the fact that no students responded “I did not watch the clinician/patient videos” on the post-course survey. Even if we were able to capture these direct measures, there can be conflict between what subjects report as engaging and what is physiologically measured as engaging [37]. What is more important is how that engagement drives behavior and impacts learning, and we currently do not know whether perceived engagement or measured physiologic engagement is a greater factor.

### Conclusion

Although we describe some benefits of the VoC videos, they are not meant to replace traditional, in-person clinical immersion programs, which have broader and deeper learning goals. Student participants in one clinical immersion program reported most frequently that they learned medical knowledge (65%), while a minority commented on patient (7%) and physician (17%) experiences and empathy (3%) [38]. Although sometimes described in the VoC videos, background on disease states, treatments, and procedures is not the focus, so students would not be expected to develop this valuable skill through watching the VoC videos alone. Instead, the focus of the VoC videos is on user experiences relevant to a specific medical device. Similar to student-reported benefits of a clinical immersion program, the VoC videos help students understand about the design of medical equipment and the needs of their users [38].

Since in-person clinical immersion programs are difficult to scale, we have addressed this difficulty by bringing the clinical and patient perspectives to the classroom through videos. The VoC videos are not meant as an instructional aid for the “capture” stage of needs finding (unless an instructor wants to use them as a tool to evaluate how useful or not various interview questions were). The VoC videos are more useful as a tool for the “synthesis/analysis” stage. In this stage, the intention is that the students watch the VoC videos, identify themes within and across the VoC videos, and compare these themes to literature or other sources. Students value hearing from different stakeholders and diverse perspectives through the VoC videos, so future work that intends to expand the VoC video portfolio should prioritize interviewing people with different experiences using these devices.

These VoC video interviews serve to ensure that all students in our program are introduced to skills in needs identification, problem definition, and empathy, which can be further built upon in latter courses and experiences in the undergraduate curriculum. Videos have the additional advantage that they can be reused in subsequent years and can scale to online platforms for distance learning, as needed.

To conclude, we have described a process to create Voice of the Customer (VoC) video interviews that serve as educational tools for the design of medical devices. Students may use these videos, available online at https://sites.udel.edu/meddevicesvoc/, to identify unmet clinical needs, define design inputs, develop empathy for medical device users, and support engagement in their course content.

## Supplementary Information

Below is the link to the electronic supplementary material.

Supplementary file1 (DOCX 37 kb)

## Data Availability

Aggregate data are available on request, pending any restrictions due to ethical considerations. Please contact the corresponding author. VoC videos are available at https://sites.udel.edu/meddevicesvoc/.
