# Supplementary material for: Voice of the Customer Videos: An Educational Tool to Identify Unmet Clinical Needs and Develop Empathy for Medical Device Users
Source: Biomed Eng Educ. 2025 Dec 2;6(1):153–69. doi: 10.1007/s43683-025-00206-5 (PMC12876067; doi:10.1007/s43683-025-00206-5)
Supplement: Supplementary file 1 — Supplementary file1 (DOCX 37 kb) [file 43683_2025_206_MOESM1_ESM.docx]

# **Appendix A**: General Interview Questions

A semi-structured interview approach was used for all voice of the customer interviews. The list below is a generalized set of questions that were then tailored for each of the six medical devices and for the different types of stakeholders being interviewed.

- Introduce yourself with your name, title, and most important responsibilities.
- Tell me (generally) the purpose of this medical device and when/why you use it.
- How do you define “success” of the device?
- What are the key components of the device, and can you demonstrate how the device works?
- How often do you use this device? For what duration of time?
- When you’re done using the device, what happens next (dispose, clean-how)?
- What features of this device do you like?
- What are some of your frustrations (challenges, obstacles, annoyances, pain points) in using this device?
- Have you ever had/seen anything go wrong with the device? How did you know? How did it get fixed?
- Are there any improvements you’d recommend?
- How did you decide to use a particular version of this medical device?
- Can you tell me about the pros/cons of other similar devices you have used?
- Do you remember the first time you learned to use the device? Can you describe that experience?
- What is the process or workflow to get/adopt/recommend a new device?
- Tell me (specifically) about a memorable story or the last time you used this medical device.
  - What was the situation?
  - Where did it take place?
  - Who was involved?
  - Why did you need to use the device?
  - How long did it take?
  - Did the experience/procedure go as planned? Why/why not?

# **Appendix B**: Rubrics Used to Score Student Team Deliverables

**Rubric to Evaluate Incorporation of VoC Videos in Need Statement Justifications**

| **Full Marks** |
| --- |
| Justifications apply Voice of Customer (VoC) insights that align completely with the need statement. |
| **4 pts** |
| **Proficient** |
| Justifications reflect stakeholder feedback/VoC and are generally tied to the need statement, though some connections could be clearer. |
| **3 pts** |
| **Developing** |
| Stakeholder feedback/VoC is reflected, but the connections to the need statement are weak or unclear. |
| **2 pts** |
| **Needs Improvement** |
| Limited use of stakeholder feedback/VoC, and connections to need statement are not well established. |
| **1 pts** |
| **No Marks** |
| No evidence of stakeholder feedback or use of the Voice of the Customer is provided. |
| **0 pts** |

**Rubric to Evaluate Incorporation of VoC Videos as Sources for Design Inputs**

| **Full Marks** |
| --- |
| Sources reflect stakeholder feedback/VoC and are effectively tied to functional or design requirements. |
| **4 pts** |
| **Proficient** |
| Sources reflect stakeholder feedback/VoC and are generally tied to the requirements, though some connections could be clearer. |
| **3 pts** |
| **Developing** |
| Stakeholder feedback/VoC is reflected, but the connections to design and functional requirements are weak or unclear. |
| **2 pts** |
| **Needs Improvement** |
| Limited use of stakeholder feedback/VoC, and connections to requirements are not well established. |
| **1 pts** |
| **No Marks** |
| No evidence of stakeholder feedback or use of the Voice of the Customer is provided. |
| **0 pts** |

# **Appendix C**: Survey Questions

## *Self-Efficacy of Engineering Design Process Skills Pre- and Post-Course Survey Questions*

[Survey questions are from Carberry AR, Lee H-S, Ohland MW. Measuring Engineering Design Self-Efficacy. Journal of Engineering Education. 2010;99:71–9.]

The next set of questions ask about self-efficacy the engineering design process.

Please answer the following questions fully by selecting the answer that best represents your beliefs and judgment of your current abilities. Answer each question in terms of who you are and what you know today about the given tasks. There are no right or wrong answers.

[In this paper, we report only on one subdimension of engineering design from the instrument, which is best aligned with the purpose of the VoC videos; however, the instrument in its entirety, as reported in Carberry et al.’s paper, was used on the administered survey.]

**Subdimension:** Identify a design need

**Scales:**

- **Rate your degree of confidence** (i.e., belief in your current ability) to perform the following tasks by recording a number from 0 to 100. (0 = cannot do at all; 50 = moderately can do; 100 = highly certain can do)
- **Rate how motivated** you would be to perform the following tasks by recording a number from 0 to 100. (0 = not motivated; 50 = moderately motivated; 100 = highly motivated)
- **Rate how successful** you would be in performing the following tasks by recording a number from 0 to100. (0 = cannot expect success at all; 50 = moderately expect success; 100 = highly certain of success)
- **Rate your degree of anxiety** (how apprehensive you would be) in performing the following tasks by recording a number from 0 to 100. (0 = not anxious at all; 50 = moderately anxious; 100 = highly anxious)

[Questions and scales were arranged in a “matrix”-style format through Qualtrics with response options from 0 to 100 by 10s.]

## *Empathy Pre- and Post-Course Survey Questions*

[Survey questions are modified from

Drouet L, Bongard-Blanchy K, Lallemand C. Development of the Empathy in Design Scale: Measuring Employees’ Empathy Toward Users in Service Design. Interacting with Computers. 2024;36:313–34.

The words that are modified from the original Empathy in Design Scale are underlined below but were not underlined in the version administered to students.]

The next questions are related to the Biomedical Engineering context.

The table below includes statements related to the Biomedical Engineering context. By ‘users’ we mean people/customers who use products (i.e., medical devices). Please use the 7-point scale to indicate the degree to which these statements accurately describe you or not **(1 = ‘Does not describe me at all’ to 7 = ‘Completely describes me’).**

**Scale:**

1=Does not describe me at all

2=Barely describes me

3=Somewhat describes me

4=Moderately describes me

5=Generally describes me

6=Mostly describes me

7=Completely describes me

**Questions:**

I am interested to learn about users’ experiences and needs.

I am curious about users’ experiences and needs.

I want to learn about users’ experiences and opinions about medical devices.

As a Biomedical Engineering student, I try to find out what the users’ needs are.

As a Biomedical Engineering student, I take action to view things from the user’s perspective.

As a Biomedical Engineering student, I actively listen to users’ experiences to better understand their perspectives.

The experiences and feelings of users resonate with my own.

I understand the users’ experiences because I know how it feels.

I am aware that my experiences as a Biomedical Engineer are different from the ones of users.

I realize that there are similarities and differences between my experiences and the ones of users.

I understand why users perceive things differently than I do as a Biomedical Engineer.

[Questions and scale were arranged in a “matrix”-style format through Qualtrics.]

## *Clinician/Patient Videos Post-Course Survey Questions*

The following questions ask about the clinician/patient videos used during the course.

To what extent did the clinician/patient video foster engagement in course content?

- Extremely engaging (5)
- Very engaging (4)
- Moderately engaging (3)
- Slightly engaging (2)
- Not at all engaging (1)
- I did not watch the clinician/patient videos

To what extent did the clinician/patient video help you identify unmet health needs of existing devices?

- Extremely helpful (5)
- Very helpful (4)
- Moderately helpful (3)
- Slightly helpful (2)
- Not at all helpful (1)
- I did not watch the clinician/patient videos

To what extent did the clinician/patient video impact your empathy for users of medical devices?

- Very strong impact (5)
- Strong impact (4)
- Moderate impact (3)
- Slight impact (2)
- No impact at all (1)
- I did not watch the clinician/patient videos

[Questions and scales were arranged in a multiple-choice style format through Qualtrics.]

# **Appendix D**: Focus Group Questions Specific to VoC Videos

In-person focus groups led by a professional external evaluator were used to collect student perspectives about the course. Although the focus groups covered many topics, here we report only on questions that relate to the VoC videos. The focus group questions slightly shifted between 2024 and 2025 to reflect that the research questions were refined over time.

## *2024 Focus Group Questions*

| **Clinician/Patient Videos** |
| --- |

*Clinician/patient videos were tools used to support your learning in the course. I would like to hear your feedback on these.*

**Overall, what did you think about these videos? [Will ask for each Unit]**

- What did you find useful/helpful?
- What (if anything) was not useful/helpful?
- What suggestions can you provide for improvements?
- How/did the voice of the customer videos help you understand the clinical context?
- How/did the voice of the customer videos help you identify unmet health needs of existing devices?
- In what way (if any) did the customer videos impact your perspective taking? Empathy?

## *2025 Focus Group Questions*

| **Clinician/Patient Videos** |
| --- |

*Clinician/patient videos were tools used to support your learning in the course. I would like to hear your feedback on these.*

**Overall, what did you think about these videos? [Will ask for each Unit]**

- What did you find useful/helpful?
- What (if anything) was not useful/helpful? Why not?
- What suggestions can you provide for improvements?
- How/did the voice of the customer videos foster engagement in course content?
- How/did the voice of the customer videos help you identify unmet health needs of existing devices?
- In what way (if any) did the customer videos impact your empathy for users of medical devices?

# **Appendix E**: Example Student Work

## **Need Statement Justifications**

The two excerpted examples of student work below both scored a 4 for applying VoC to the justification of their need statement. Redacted interviewee names are replaced with [XXX].

### *Example Student Work 1*

Need Statement:

A way to improve the weight and size of surgical staples for medical professionals (nurses, physician assistants, doctors, etc.) to increase usability between different-sized medical professionals.

Explanation & Justification (include voice of customer and support with additional references):

Surgical staplers are used by different clinicians in the operating room for a variety of different procedures. Many different clinicians have trouble using these staplers as they are not made for all users of the device [student reference 1]. Some users also state how fatigue plays a role in surgery due to their use of surgical staplers [student reference 2]. According to Dr. [AAA] staplers are big when they are being handled in the operating room [student reference 1]. Additionally [BBB] states how fatigue affects her use of the stapler [student reference 3]. Lastly, Dr. [CCC] notes how Bariatric surgeons can have trouble stapling the stomach due to fatigue [student reference 9]. Creating a more usable device for users will allow for more efficient and safe surgeries for both patients and clinicians/users.

Voice of customer and additional references:

1). [AAA] Quote: “You know, the biggest con right now is the size of these staplers. We’re working with these like tiny, thin little phones that can do so much and our Apple Watches and everything else- they’re all compact in there. And yet we have staplers that are pretty big that we have to handle.”

[…]

3). [BBB] Quote: “Sometimes when you go to pop the cartridge out, your thumbs are so sore sometimes. It would be nice if you could just pop it out because sometimes you gotta put on your table and pop it out like that.”

[…]

9). [CCC] Quote: “I can fire my staplers by hand faster, and power doesn’t provide me any benefit. The power it provides I think the bariatric surgeons benefit because they’re getting tired, their hands get a little weak trying to staple that stomach.”

### *Example Student Work 2*

Need Statement:

A way to improve how the Continuous Glucose Monitor attaches to a patient's skin in order to prevent unnecessary damage to the area of attachment due to the adhesive and minimize the number of unnecessary devices needed in a time period.

Explanation & Justification (include voice of customer and support with additional references):

CGM users experience their monitor falling off from sweating, force, or other external factors. User [DDD] mentions how during the summer months, the monitor would fall off due to sweating, a statement. Another user, [EEE], how hers got ripped off by her baby brother. This sentiment is echoed by many other users of a Continuous Glucose Monitor. The National Library of Medicine even has an article about this issue, stating that “Device adhesive issues included transmitter and sensor fall-offs, accidental pull-offs, and transmitters becoming uncoupled from the sensor.” [student reference 1] Another article in this library truly summarizes the effects of this issue, stating “If the CGM sensor falls off, then it will need to be replaced, further increasing the burden for people with diabetes.” [student reference 2]

[1] K. Englert et al., “Skin and adhesive issues with continuous glucose monitors,” Journal of Diabetes Science and Technology, vol. 8, no. 4, pp. 745–751, Apr. 2014, doi: 10.1177/1932296814529893.

[2] T. Tian et al., “Use of continuous glucose monitors upon hospital discharge of people with diabetes: promise, barriers, and opportunity,” Journal of Diabetes Science and Technology, vol. 18, no. 1, pp. 207–214, Oct. 2023, doi: 10.1177/19322968231200847.

## **Design Input Sources**

The two excerpted examples of student work below both scored a 4 for applying VoC as sources for their design inputs. Only a subset of the design criteria is provided for each example because submissions were extensive. Redacted interviewee names are replaced with [XXX].

### *Example Student Work 1*

| Source | Functional Requirement | Design Requirement |
| --- | --- | --- |
| [FFF] “the CGM devices that are available, they sort of just surface raw data” [student reference]  --------------------------------------  [GGG] “One of the things that Dexcom does not do for you and that I had to find some other way to do was to do standard deviations.” [student reference] | Deeper data analysis | The display device must show current glucose levels and trends over time to determine a trendline for blood glucose concentrations |
| [HHH] “I like that my Dexcom is smaller” [student reference] | Small dimensions | Mass: <5 grams, Radius: <15mm, Thickness: <5mm |
| [III] “Occasionally, particularly in the summer months, when people sweat a lot, the adhesive gets loose and would fall off” [student reference] | Stickier, non-allergenic adhesive that is also easy to remove (sweat resistant) | An adhesive that is unlikely to cause skin irritation for the minimum to maximum  lifespan of the device (7-14 days) while remaining firm and keeping the device intact |
| [JJJ] “Now with the Dexcom, I don’t have to do any moving, really. It just automatically sends it to my pump...” [student reference] | Compatible and automatic communication  with multiple brands of pumps | Contains a Bluetooth device that can wirelessly communicate data between insulin pumps, the CGM itself and any third party tracking device |
| [III] “They really are designed to be, you know, the type of glucose sensor that you can self-insert. So I think that’s also something that’s very important to consider when they’re glucose sensors because we want people to use this on their own and not rely on healthcare providers to put it on for them.” [student reference] | Easy self-insertion process | The ability to insert the device from one push of a button and the entire apparatus taking  only one person to apply properly |

### *Example Student Work 2*

| Source | Functional Requirement | Design Requirement |
| --- | --- | --- |
| According to Dr [LLL] one of the challenges of working with stents is choosing the correct length to ensure the entire afflicted area is covered, “Challenging parts is choosing the appropriate length of the stent to make sure you’re covering the entire disease segment.” [student reference] | The stent length must be adjustable whether autonomously or through laparoscopic adjustment in order to ensure the entire  portion of afflicted blood vessel is covered. | Stent length can vary from 8 mm to 70 mm in diameter, the stent should be adjustable for an  additional 6 mm in length on each end of the stent. |
| According to Dr. [MMM], “A key design challenge is developing stents that can be re-dilated 3–5 times their original diameter.” [student reference] | The stent must minimize long-term complications and repeat procedures,  especially in pediatric patients and underserved populations. | Stent must be redilatable 3–5× within 2 mm–3 mm its original diameter to support and  accommodate vessel growth and avoid repeated interventions. |
| According to Dr. [NNN], “The body acts aggressively against any foreign body... there is a fibrotic layer that happens inside the stent.” [student reference] | The stent must avoid triggering immune or  cultural rejection due to material composition. | The stent must exclude animal-derived components (e.g., bovine gelatin) and use only synthetic or plant-based polymers. |
| According to Dr. [LLL], “You want a stent that is easily trackable... deliverable, so it can go to any vessel where you desire to deploy the stent.” [student reference] | The stent delivery system must be optimized for trackability and deployment precision in a wide range of clinical settings | The stent shall be visible under low-resolution fluoroscopy and deliverable through ≤6 Fr  catheters with guide extension compatibility. |

# **Appendix F**: Modified Empathy in Design Scale Item Ratings

|  | **Pre** | **Post** | **Change** |
| --- | --- | --- | --- |
| **Emotional interest and Perspective taking** | 5.22 (0.88) | 5.79 (0.72) | 0.57*** |
| I am interested to learn about users’ experiences and needs. | 5.06 (0.95) | 5.79 (0.81) | 0.73*** |
| I am curious about users’ experiences and needs. | 5.24 (0.96) | 5.68 (0.91) | 0.44* |
| I want to learn about users’ experiences and opinions about medical services. | 5.15 (0.89) | 5.65 (0.98) | 0.50** |
| As a Biomedical Engineering student, I try to find out what the users’ needs are. | 5.12 (1.01) | 5.91 (0.87) | 0.79*** |
| As a Biomedical Engineering student, I take action to view things from the user’s perspective. | 5.26 (1.11) | 5.82 (0.80) | 0.56* |
| As a Biomedical Engineering student, I actively listen to users’ experiences to better understand their perspectives. | 5.47 (1.08) | 5.91 (0.83) | 0.44* |
| **Personal Experience** | 4.47 (1.40) | 4.76 (1.29) | 0.29 |
| The experiences and feelings of users resonate with my own. | 4.58 (1.42) | 5.06 (1.17) | 0.48* |
| I understand the users’ experiences because I know how it feels. | 4.36 (1.50) | 4.45 (1.54) | 0.09 |
| **Self-Awareness** | 5.36 (1.08) | 6.10 (0.65) | 0.74*** |
| I am aware that my experiences as a Biomedical Engineer are different from the ones of users. | 5.24 (1.30) | 5.94 (0.89) | 0.70** |
| I realize that there are similarities and differences between my experiences and the ones of users. | 5.45 (1.09) | 6.15 (0.83) | 0.70*** |
| I understand why users perceive things differently than I do as a Biomedical Engineer. | 5.39 (1.17) | 6.21 (0.74) | 0.82*** |

Values are means (standard deviation) on a 1-7 scale. *p<0.05; **p<0.01; ***p<0.001, N = 36

[Survey questions are modified from Drouet L, Bongard-Blanchy K, Lallemand C. Development of the Empathy in Design Scale: Measuring Employees’ Empathy Toward Users in Service Design. Interacting with Computers. 2024;36:313–34.]
